# Health Risk Factors, Prevention, and Inequalities

**DOI:** 10.3390/medicina61010127

**Published:** 2025-01-14

**Authors:** Pietro Ferrara

**Affiliations:** 1 Center for Public Health Research, University of Milan–Bicocca, 20900 Monza, Italy; pietro.ferrara@unimib.it; 2 Laboratory of Public Health, IRCCS Istituto Auxologico Italiano, 20149 Milan, Italy

## 1. Foreword

The powerful quote from Martin Luther King Jr. “*Of all the forms of inequality, injustice in health care is the most shocking and inhumane*” (1966) highlights the critical importance of addressing disparities in health as a matter of social justice and underscores the need for equitable access to care. Spoken over half a century ago, these words resonate with striking relevance today. As we face a world where health risks are unevenly distributed, prevention efforts fall short for the most vulnerable people, and inequalities persist despite advances in medicine, his insights serve as both a challenge and a call to action [1,2].

Health is a fundamental pillar of human well-being, influencing the quality and longevity of life and the overall economic and social development of populations. However, the attainment of optimal health is neither universally accessible nor equitably distributed. This disparity stems from a complex interplay of risk factors, preventive measures, and structural inequalities that shape health outcomes across different populations. Addressing these challenges requires a comprehensive understanding of the dynamics between individual behaviors, environmental conditions, and systemic barriers to health equity [2,3,4].

Health risk factors—such as the use of tobacco products, poor or excessive nutrition, physical inactivity, and harmful use of alcohol and other substances—are well established contributors to the global burden of disease. Non-communicable diseases (NCDs), including cardiovascular diseases, diabetes, chronic respiratory diseases, and cancers, account for more than 70% of deaths worldwide, and many of these are linked to modifiable behaviors. Moreover, communicable diseases, despite significant progress in prevention and treatment, remain prevalent in low- and middle-income countries, where access to healthcare services is limited. The convergence of these risk factors often amplifies the challenges for vulnerable populations, exacerbating pre-existing inequalities [5].

Prevention lies at the heart of public health efforts to mitigate the impact of these risk factors. Primary prevention strategies—including vaccination, education on healthy lifestyles, and environmental interventions—aim to reduce the incidence of disease by addressing its root causes. Secondary and tertiary prevention measures focus on early detection and effective management of diseases to minimize complications and improve quality of life. Despite the proven efficacy of preventive measures, their implementation often falls short due to resource constraints, insufficient policy support, and social determinants of health that limit access and uptaking among disadvantaged groups [6,7].

Inequalities in health outcomes are deeply entrenched within social, economic, and cultural contexts. Factors such as income, education, gender, ethnicity, and geographic location significantly influence an individual’s ability to achieve and maintain good health. The COVID-19 pandemic underscored these disparities, as marginalized communities experienced disproportionately higher rates of infection, severe illness, and death. Structural inequities, including inadequate healthcare infrastructures, discriminatory practices, and systemic bias, further perpetuate these gaps, challenging efforts to promote health equity [2,3,8,9].

In this Special Issue, the authors explored the interconnected themes of health risk factors, prevention, and inequalities, emphasizing the need for a multidimensional approach to tackling these issues. By proposing original research, highlighting innovative interventions, and advocating for policy changes, the collected evidence inspires actionable strategies that prioritize prevention and equity in health. Achieving sustainable progress in health on a global scale demands not only addressing the immediate challenges of risk factors and diseases but also dismantling the systemic barriers that prevent equitable access to health and well-being for all [10].

## 2. Special Issue Overview

Thirty-six articles were submitted for consideration for the Special Issue and underwent rigorous evaluation by editors and reviewers as part of the *Medicina* review process. Of these, 11 papers were finally accepted for publication and inclusion in this Special Issue. These can be accessed at the Special Issue webpage, and a brief description of the contributions follows below.

The first contribution, by Mognetti et al., explored the relationship between alcohol consumption and sexual violence, analyzing the prevalence, characteristics, and consequences of such incidents. The findings indicate that alcohol-consuming victims were generally younger, predominantly aged 18–25, and assaults involving them were more likely to occur in public places or in someone else’s home. Perpetrators were often acquaintances or unknown individuals for alcohol consumers, whereas partners were the most common assailants for non-alcohol consumers. These insights can inform targeted interventions and prevention strategies to address sexual violence, considering factors such as nationality, age, and assailant identity that influence the dynamics of such incidents.

Ponticelli et al. examined the awareness and perspectives of Italian cardiologists and cardiac nurses regarding the recombinant zoster vaccine (RZV). The study found that only 29.1% of healthcare professionals correctly knew the RZV schedule, and 57.6% were aware of its suitability for immunocompromised individuals. Factors such as higher education levels, participation in vaccination updates, and awareness of herpes zoster complications were associated with better knowledge. This work highlighted the need for targeted educational initiatives to improve understanding and the uptaking of RZV among cardiac healthcare providers in Italy.

The research by Zalewska et al. studied women’s knowledge of reproductive health issues, highlighting gaps in understanding and emphasizing the need for enhanced educational initiatives to improve health outcomes.

Schauer et al. examine differences between male and female bank employees in terms of health indicators, lifestyle behaviors, and nutritional status. This research contributes to addressing gaps in the understanding of health dynamics in adult populations with sedentary occupations and underscores the specific needs of male and female employees to promote overall well-being in the workplace.

The contribution by Huang et al. analyzed the association between sociodemographic characteristics, health-related factors, and sedentary behavior among Taiwanese adults aged 50 and above. Findings indicate that higher sedentary times are significantly associated with being male, older ages, lower education levels, poorer self-reported health, and lower physical activity. These results suggest the need for specific interventions to reduce sedentary behavior, particularly among high-risk groups, to promote healthier lifestyles in middle-aged and older populations.

Pradipta et al. documented the prevalence and factors associated with treatment nonadherence in patients with multiple chronic conditions. Analyzing data from the Indonesian Family Life Survey, the researchers found that 36.4% of multimorbid patients were nonadherent to their treatment regimens. Factors significantly associated with nonadherence included good self-perceived health, active smoking behavior, lack of insurance ownership, lower income, and smaller household size. With their work, authors indicated the need to consider creating patient-tailored treatment programs to improve treatment adherence among multimorbid patients in Indonesia.

Utilizing data from the Serbian National Health Survey, Dimitrijev et al. identified significant associations between elevated blood pressure (prehypertension and hypertension) and factors such as age, gender, education level, employment status, and lifestyle behaviors. The findings indicate that older age, male gender, lower education, unemployment, and unhealthy lifestyle choices are linked to higher risks of prehypertension and hypertension. These results underscore the importance of targeted public health interventions focusing on modifiable risk factors to address the growing burden of hypertension in Serbia.

To learn how prenatal education influences adherence to World Health Organization (WHO)-recommended prenatal practices among Ngäbe–Buglé women, Johnson et al. surveyed 137 women, assessing their compliance with practices such as taking prenatal vitamins, attending check-ups, avoiding alcohol, increasing caloric intake, and maintaining activity levels. Findings revealed that only 47% took prenatal vitamins, 15% attended prenatal check-ups, and 23% increased caloric intake, while 99% avoided alcohol and 74% maintained activity levels. Significantly, women who received a prenatal education from official medical providers demonstrated higher adherence to taking prenatal vitamins compared to those educated through unofficial sources or not at all. Based on their results, the authors speculated that culturally competent prenatal education and improved access to healthcare services are key to enhancing adherence to recommended prenatal practices in these communities.

Gammoh et al. estimated the prevalence of severe fibromyalgia, depression, anxiety, and insomnia symptoms among Arab women and explored the potential link to self-medication with analgesics. The research reveals a significant association between self-medication and increased severity in these symptoms, highlighting a concerning trend in this population. While public health interventions are crucial to addressing self-medication practices and promoting appropriate medical consultation, the study shed lights on the importance of mental health support and education to mitigate the risks associated with unmanaged psychological symptoms.

Chen C. et al. assessed the prevalence of frailty among older adults in Taiwan and identifies associated demographic, physiological, and functional factors. Utilizing the Kihon Checklist (KCL), researchers assessed 278 community-dwelling older adults, categorizing them as robust, prefrail, or frail. The findings revealed that 12.2% were frail, 45.7% prefrail, and 42.1% robust. Factors significantly associated with frailty included advanced age, lower education levels, poor nutritional status, decreased muscle strength, and reduced physical performance. Early frailty identification and classification, as well as targeted interventions aimed at improving lower-limb strength, endurance, and mobility, are essential to preventing and delaying frailty progression in older adults.

In the last contribution, Chen Y. et al. found that married healthcare workers exhibited a higher prevalence of prediabetes compared to their unmarried counterparts. A further analysis indicated that elevated triglyceride levels partially mediated this relationship, suggesting that marriage may influence prediabetes risk through its impact on lipid metabolism. These findings highlight the importance of monitoring triglyceride levels and implementing lifestyle interventions to mitigate prediabetes risk.

## 3. Conclusions

While the topics discussed in the articles of this Special Issue are diverse and heterogeneous, they collectively contribute to advancing and stimulating the scientific debate on health risk factors, prevention, and inequalities. Addressing these interconnected challenges requires a holistic and inclusive approach centered on equity and sustainability. Together, the contributions in this Special Issue serve as a unified call to action, urging stakeholders across sectors to collaborate in creating a healthier and more equitable world. By leveraging innovative interventions, evidence-based strategies, and policy reforms, we can work towards bridging the gaps in health outcomes and ensuring that no one is left behind.
